# How do clinical genetics consent forms address the familial approach to confidentiality and incidental findings? A mixed-methods study

**DOI:** 10.1007/s10689-017-9994-9

**Published:** 2017-04-12

**Authors:** Sandi Dheensa, Gillian Crawford, Claire Salter, Michael Parker, Angela Fenwick, Anneke Lucassen

**Affiliations:** 1 Clinical Ethics and Law, Faculty of Medicine, Southampton General Hospital, University of Southampton, Room AB 203, MP 801, South Academic Block, Tremona Road, Southampton, SO16 6YD UK; 2 Wessex Clinical Genetic Service, University Hospital Southampton NHS Foundation Trust, Southampton, UK; 3 The Ethox Centre, Institute of Health Sciences, University of Oxford, Old Rd, Oxford, OX3 7LF UK

**Keywords:** Clinical ethics, Consent, Confidentiality, Genomics

## Abstract

Genetic test results can be relevant to patients and their relatives. Questions thus arise around whether clinicians regard genetic information as confidential to individuals or to families, and about how they broach this and other issues, including the potential for incidental findings, in consent (forms) for genetic testing. We conducted a content analysis of UK-wide genetic testing consent forms and interviewed 128 clinicians/laboratory scientists. We found that almost all genetic services offered patients multiple, sometimes unworkable, choices on forms, including an option to veto the use of familial genetic information to benefit relatives. Participants worried that documented choices were overriding professional judgement and cautioned against any future forms dictating practice around incidental findings. We conclude that ‘tick-box’ forms, which do little to enhance autonomy, are masking valid consent processes in clinical practice. As genome-wide testing becomes commonplace, we must re-consider consent processes, so that they protects patients’—and relatives’—interests.

## Introduction

Several issues complicate the process of seeking consent in clinical genetic practice, including the potential for tests to produce ‘incidental findings’ and the fact that test results might indicate that the patient’s family members are at risk. In this paper’s introduction, we start by examining these two issues and the way they relate to the consent process, before introducing some considerations about the way consent forms might help healthcare professionals (HCPs) to tackle the issues.

Broad genome tests can produce a range of findings unrelated to the clinical reason for doing the test. These findings might be actively sought (although this is not yet offered in routine clinical practice in the UK) or they might be discovered ‘incidentally’ in the search for a diagnostic variant. We refer to these latter findings in this paper as incidental findings (IFs), although various other terms are used [1]. Tests might also produce findings that have unclear clinical meaning: these are called variants of uncertain/unknown significance (VOUS). On the question of whether and how IFs should be addressed in the consent process, some commentators have suggested giving patients categories of IFs and asking them to decide which they would want to know about [2, 3]. Others retort that IFs cannot be accurately categorised and that consent to the general possibility of receiving clinically significant information is possible [1]. The potential for so many different findings means that consent in this regard can only ever be broad, and not, as desired by clinical and research governance frameworks, ‘fully informed’ [1].

Whether a primary or incidental finding, genetic test results can be relevant to the patients’ family members. They might indicate that relatives are at risk, and IFs might require relatives to undergo testing to determine the finding’s clinical significance [1]. Thus, when a genetic test is carried out, multiple interests are at stake, which raises questions about how consent should proceed, given that it usually embodies respect for *individual* autonomy. There are further questions about how HCPs should conceptualise and discuss confidentiality in the consent process. The usual medical view is that all information should be kept confidential to the individual. By contrast, the familial view of confidentiality proposes that HCPs treat personal clinical information (e.g., a diagnosis of breast cancer) as confidential to the individual, but treat genetic information (e.g., a BRCA1 mutation) as confidential to the family and use this information to benefit the health of known at risk relatives [4]. By adopting this approach, i.e., by separating the personal clinical information from the familial genetic information, a HCP who becomes aware of an at-risk relative can offer them an accurate test, without having to recontact the original patient for permission. It also means an individual patient is not given veto power over the sharing of familial information. Guidance published by the UK Joint Committee on Genomics in Medicine (JCGM: 2011) [5] encourages HCPs to take this approach and to make it explicit to patients during the consent discussion.

### Using forms to document consent

Given these complexities, it is worth asking whether consent forms can help HCPs to seek consent and what these consent forms should mention. Consent in clinical practice is usually implied and integrated into clinical discussions, although UK guidelines suggest it should additionally be in writing if it is for a complex test and/or one that has potentially significant consequences for the patient’s life [6]. By contrast, consent for research, which is underpinned by international guidelines and declarations (e.g., the Declaration of Helsinki), is generally a formalised, detailed, and written process that happens before research activity begins [7–9]. The complexity of next-generation tests, such as whole-genome sequencing, have led some authors to suggest that clinicians should use consent forms that more closely resemble research forms, i.e., ones that cover a range of topics, in routine clinical practice [2]. In clinical genetics, the line between clinical practice and research is sometimes blurred (for example, in the UK, patients can get a clinical test result from participating in research studies or ‘hybrid’ clinical-research ventures, such as the 100,000 genomes project [10]).

**Fig. 1:**
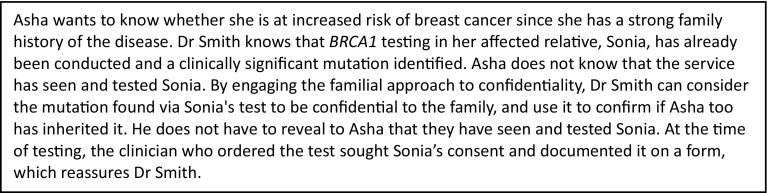
Clinical scenario to illustrate the familial approach

**Fig. 2:**
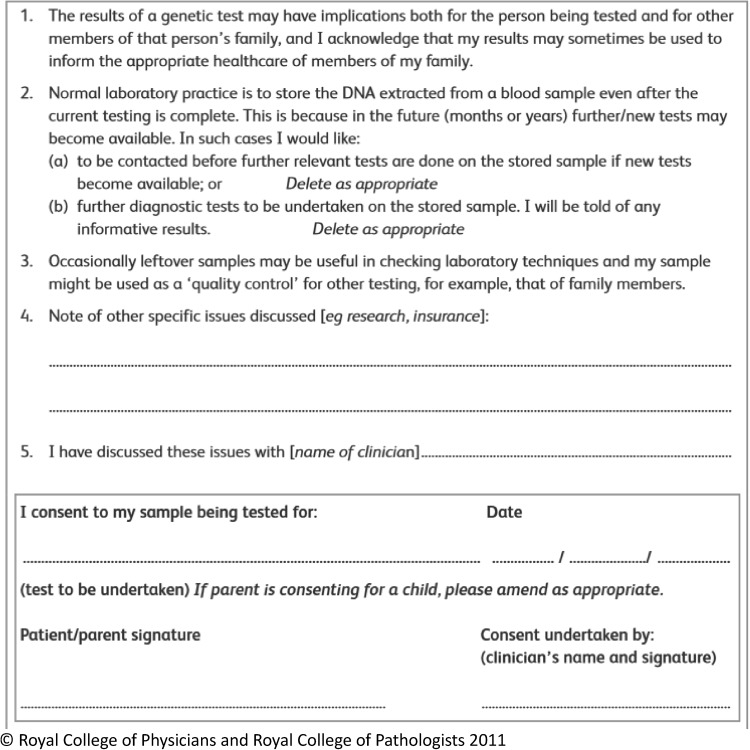
JCGM form [5]

In their 2011 guidelines, the JCGM recommended a ‘template’ consent form for use in clinical genetic practice. The template is kept deliberately simple, unlike a research consent form, and includes just four key statements, which correspond to aspects of practice that the committee argue are necessary for the running of an effective genetic service. One statement is, “I acknowledge that my results may sometimes be used to inform the appropriate healthcare of members of my family.” This statement thus prompts HCPs to adopt, and make explicit to patients that they are adopting, the familial approach to confidentiality. The form does not mention IFs or VOUS explicitly and the JCGM has not endorsed providing categories of IFs from which patients can choose. It has an area for free-text where HCPs can record discussion of these findings. Figure 1 depicts the form and the case in Fig. 2 shows an example of how it could facilitate the testing of family members. Use of the form is not mandated, but the JCGM intended it to help set standards for seeking consent. The form and guideline is due for revision this year.

Interestingly, Fowler [11] found that just 11% of USA consent forms for whole-exome sequencing (WES) mentioned that test results could be relevant to relatives, despite national guidelines stating that all forms should include this point. It is thus unclear whether and how USA HCPs address this issue in the consent process for WES.

No one has examined the consent forms used for genetic tests across the UK, although there is research about HCPs’ views about IFs [1] and about familial confidentiality [12]. Examining consent forms would be valuable because forms prompt and guide discussion in the consent process [13]. The content of forms can thus shed light on the way different clinics tackle consent, the way HCPs approach confidentiality, and the way they manage IFs.

Investigating clinical consent processes in general is worthwhile to see whether a concern that Dunn [14] has expressed bears out in genetic practices. He opines that consent is losing its moral and theoretical underpinnings and is becoming an “overly proceduralised...tick-box exercise that exists solely to ensure that medical practice is aligned with prevailing professional requirements” (p. 67).

We therefore conducted a systematic investigation into the way UK genetic services are seeking and documenting consent for genetic tests in current practice. We did this by analysing the content of consent forms and by conducting interviews/focus groups, in which HCPs discussed using (or not using) them. We paid attention to whether and how forms mentioned benefit to relatives, and thus their implied approaches to confidentiality, and whether and how they mentioned IFs. In addition, we examined a more general issue: whether the forms comprised of statements or, as Dunn worries, multiple choices and tick-boxes that might undermine consent. We make recommendations for practice in light of the upcoming revision to JCGM guidelines. Despite our specific frame of reference being guidance from the UK, our questions around consent, confidentiality, and IFs are relevant to all countries whose health services incorporate genetic testing.

## Methods

### Setting

The UK has 23 regional genetic services and 1 separate cancer genetic service, which together, serve the entire UK population (~64.6 million). Familial cancer risks are one of the most common conditions for which genetic tests are ordered.

### Design

Our study adopted a convergent parallel mixed-methods (quant–qual) design. The content analysis of consent forms was quantitative. Exploration of HCPs’ views, practices, and experiences regarding consent and the use of forms was qualitative. Analyses were conducted in parallel and were drawn together to form conclusions [15].

### Procedure

#### Quantitative content analysis

**Fig. 3:**
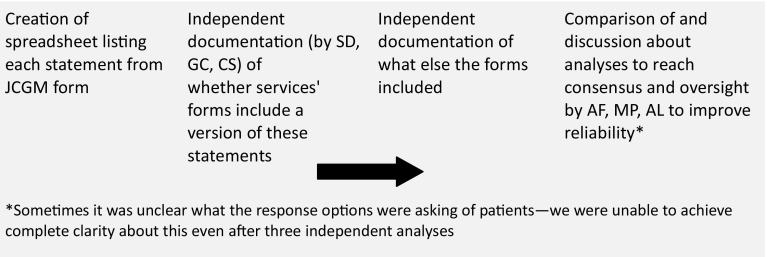
Content analysis process

We requested and received consent forms used for genetic tests from all 24 services. Where services used two or more forms, for example for adults and children, as a few did, we included both in our analysis. Analysis was an inductive process, in that we did not aim to test a particular hypothesis or theory. Due to the particularity of our documents, we did not use any specific type of content analysis. Rather, we conducted our analysis through a method (outlined in Fig. 3) most suited to the task. We identified how individual services’ forms mentioned benefit to relatives (and whether implied approaches to confidentiality were familial), the way forms mentioned IFs, and the extent to which the content and layout of forms was similar to that of the JCGM form^1^ (i.e., mentioning four key issues as statements without tick-boxes). We also considered the possible ethical and practical implications of any differences between services’ forms and the JCGM form.

#### Qualitative thematic analysis

Drawing on framework analysis [16] and a thematic analysis method [17], we examined HCPs’ perceptions of the ways that forms facilitated, constrained, or did nothing to enhance, good practice, especially regarding benefit to relatives and making decisions about IFs.

### Sample size

This was a secondary analysis: data came from two original projects, led by the current authors, which had a combined sample size of 128. The first (led by SD, AF, and AL) explored consent and confidentiality in genetic medicine and used in-depth focus groups (n = 80 across 16 groups), which SD conducted. The second (led by GC, AF, and AL) investigated consent and disclosure practices around IFs from genetic tests and used in-depth, face-to-face, interviews, which GC conducted. Our interview schedule and topic guide were based on existing literature and were semi-structured. Table 1 contains the relevant questions from both studies. They were broadly similar, e.g., they asked about practices, ethical dilemmas, and views about guidelines and HCP education. Data collection for both took place between 2013 and 2015.

**Table 1:** Interview schedule and topic guide

IFs study interview schedule	Consent and confidentiality study topic guide
• What sort of genetic tests are you organising and what range of results will these give? • How often do you order genetic tests? • What are the lab protocols for reporting results? • What are your ‘usual’ processes of seeking consent for genetic tests? • What is your experience of gene panels and how do you seek consent for these types of tests? What do you include/exclude in this discussion? • If consent is sought by non-geneticists, are there any issues this raises? • What is your understanding of the term IF? • Can you give any examples from practice where IFs have been discovered? What happened in these cases? • Are you aware of any available guidelines to help direct practice? • How do you see this issue developing in the future as genetic technologies continue to develop? • What measures would you like to see/ find useful to assist in your practice? • Is the issue of managing IFs being addressed in training of HCPs?	• What is your role? • What kinds of patients do you see? How many per week? • What other departments do you work with? Is confidentiality important in the area of medicine that you are working in? • Why do you think it is important from a patient’s point of view? • And why from a healthcare professionals’ (HCPs’) perspective? What aspects of the medical consultation should be kept confidential? • Probe about personal versus familial genetic information • Probe about confidentiality in genetic medicine versus other areas of medicine Are there guidance documents or protocols you follow for confidentiality? • What do they say? • Are they widely read? • Do people agree with them? What does/should the consent process involve when a person has a genetic test? • Is there an official consent process in your department? • How, if at all, do you talk about the limits of confidentiality in the consent process? • What do you consider these limits to be? Have you ever had a patient tell you that they were not going to inform their family of a risk? Or been unsure whether a patient had told? • To what extent do you feel like you have a responsibility to ensure patients’ family members know their risk? • What, if any, limits does this responsibility have? Regarding these issues, do you feel like you have enough support and training? Who do you talk to about ethical issues?

### Recruitment

We recruited participants from the majority of UK genetic services and a range of affiliated services. Sampling was purposive: we sought participants who ordered targeted genetic and genome tests and so were in a position to seek consent from patients. Participants were genetic counsellors, consultants, and registrars (n = 97), clinical laboratory scientists (n = 18), paediatricians (n = 7), fetal medicine specialists (n = 4), a nephrologist, and an adult physician. Participants’ names are replaced with identification codes, e.g., P1 (Participant 1 from the interview study) and FG1P1 (Participant 1 from Focus Group 1). We piloted our interview schedule and topic guide in the first interview/focus group.

### Analysis

SD and GC led the analysis. Analysis was iterative in that it involved moving back and forth between repeatedly reading transcripts, coding data, and creating themes. More specifically, we went through the data line by line and labelled salient features with codes. We collated codes into themes and then reviewed the themes to ensure they accurately reflected the codes [17]. Ongoing analysis helped us to refine the themes and the overall ‘story’ of our data. The remaining authors analysed segments of the data and critically reviewed our thematic story to improve the rigour—specifically the credibility and confirmability—of our analysis [18].

### Findings

#### Quantitative content analysis

##### (How) do genetic services’ forms mention the use of test results to benefit relatives?

**Fig. 4:**
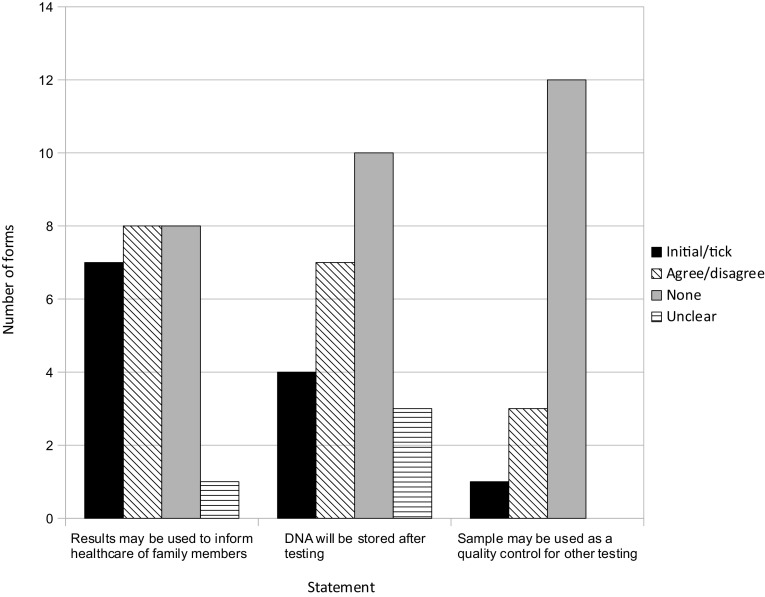
Number of services’ forms that included the JCGM statements and the response options that services provided

All UK genetic services had a consent form they could use for genetic testing, but only one used the JCGM template. Figure 4 summarises the number of services’ forms that mentioned benefit to relatives (as well as the other two topics on the JCGM form: future testing e.g. for quality control and DNA storage).

While all services’ forms mentioned the familial nature of test results, the majority enabled patients to choose whether HCPs could use genetic information to benefit their health. This finding links to a broader difference between these forms and the JCGM template: while the latter lists *statements* corresponding to practices that are argued to be necessary for the running of an effective genetic service, all services’ forms, except one, asked patients to make agree/disagree *choices* about these aspects of practice or asked for an initial or tick, the absence of which could be intended or interpreted as disagreement. This arguably reflects that UK genetic services were mostly using a ‘tick-box’ approach on consent forms. Moreover, it reflects that most services’ approach to confidentiality was individual, in that their forms gave individual patients control over genetic information, even though it might have familial implications.

##### (How) do forms mention IFs?

Unlike the JCGM form, 6/24 explicitly mentioned the possibility of IFs and of these, 5 mentioned the possibility of VOUS. There were no response options—i.e., they did not ask patients to choose whether they wanted to receive them and no forms presented patients with categories of IFs. Most forms (18/24) included a free-text area, where HCPs might record other discussions, including about these findings.

##### Aside from the JCGM statements, what else do forms mention?

We found that 23/24 of forms used in practice featured statements additional to those on the JCGM form, the most common being to ask the patient to name someone to receive their result if they are unable to do so (e.g., if they die). The number of such statements ranged from 1 to 10 (median 2.5). Table 2 lists these items with the number of services that included them. Unless otherwise stated, they had no response options.

**Table 2:** Other statements on services’ forms

Statement	No. of services that included them (and response options)
Opportunity to name an individual to receive result if patient is unable	9
Patient can change mind about receiving results	8
Understand sample will be used anonymously to develop new test^a^	7 (one gave choice)
Choice whether to receive results	6
Confirmation that written information has been given	6
Confirmation that patient understands the implications	5
Confirmation that patient has been given opportunity to ask questions	4
Option to not allow General Practitioner or speciality doctor to obtain result	4 (all gave choice)
Name of interpreter	3
Possibility for “no answers to be found” from the genetic test	3
Understand sample will be used anonymously for research	2 (one gave choice)
Understand may be contacted for research studies	2
Possibility that testing could impact insurance premiums	2
Choice of route to receive results (e.g. phone, clinic, letter)	2 (both gave choice)
Understand result will be part of electronic medical record	1

^a^The meaning here was unclear: five services included it as well as a statement about quality control

Before moving on to our qualitative analysis, it is worth noting that some of these statements were problematic. For example, asking for a named person might create difficulties if HCPs feel they should use the result for a different relative to the one named. It might also be unworkable if the named person is unreachable. Furthermore, giving patients the choice about sharing their result with their general practitioner (GP) could impede their care, since the GP is most likely to be involved in ongoing clinical management following the result. Another issue is that confirmations of receiving information and having the option to ask questions are directly drawn from research consent forms. These items relate to standard parts of clinical genetic practice. It is thus questionable whether there is any benefit in including them on consent forms. Arguably, the items are superfluous.

#### Qualitative thematic analysis

**Fig. 5:**
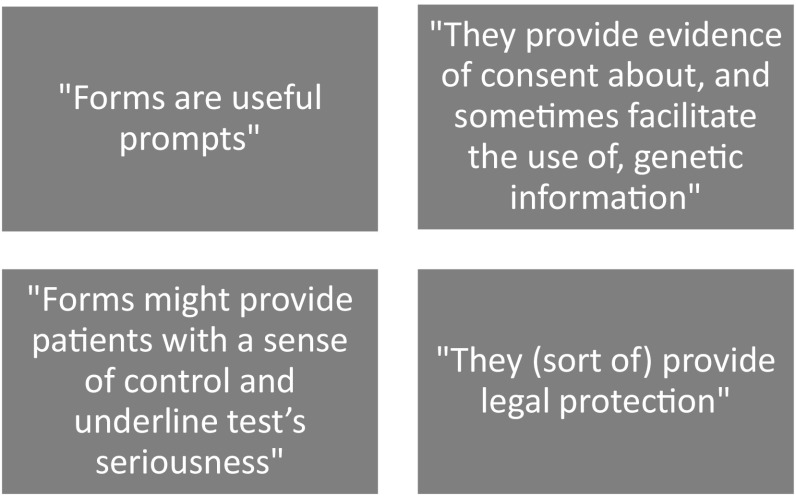
Summary of the way forms facilitated good practice

**Fig. 6:**
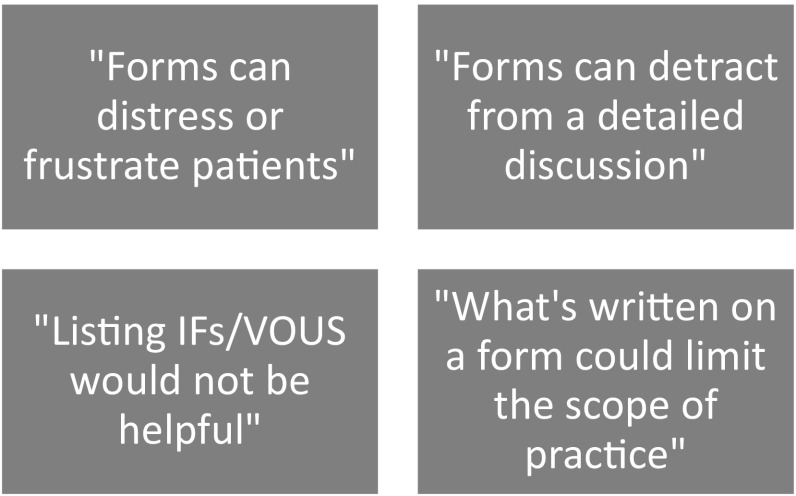
Summary of the way forms constrained, or had limited use in facilitating, good practice

Here, we present our findings from our interview and focus group studies. The findings shed light on HCPs’ views about the use of forms in general, as well as on the specific issues we have focused on so far (confidentiality and the familial nature of test results, the potential for IFs, and the tick-box style of forms). Our findings are organised into two broad themes: how HCPs thought forms (i) facilitated good practice and (ii) constrained, or had limited use in facilitating, it. Figures 5 and 6 summarise the issues raised within these themes.

##### HCPs views on how forms facilitated good practice

In this section, we outline the overlapping ways that forms were perceived to be helpful: they structured the consent discussion, potentially helped patients reflect on the testing process, and made clear to other HCPs what decisions had been made, for example around using test results to benefit relatives.

First, it merits noting that some HCPs said they did not (always) use forms, and instead inferred consent from the patient putting out their arm for the test (FG3P1; FG11P2; P31). These HCPs thought the distinction between genetic and other blood tests, for which consent forms are not used, was arbitrary, and pointed out that guidelines did not *mandate* the use of forms. By contrast, other HCPs did not consider inferred consent to be valid (FG7P2; FG9P6) and said when forms were not used (reportedly more common when a ‘mainstream’ clinician had ordered the test), it was more difficult to know what could be done with the DNA sample or genetic information;

FG12P6 “[Some clinicians] weren’t really aware consent was needed. [Mainstreaming means this has] become more of a problem recently.”

Most HCPs thought forms were useful for several reasons. One was that they prompt and focused discussion and that they enabled them to document numerous issues simultaneously (P27). HCPs moreover thought that forms helped patients to understand the potential seriousness of the test’s implications (FG15P5) and thought that having something to sign would endow a “sense of control” (P14) and some “ownership” of the decision to have the test (FG8P2).

P26 “People stop and think this is a different type of test to others, because you don’t normally sign a consent form when you have a blood test.”

FG15P5 “The number of boxes they have to read and initial might encourage people to think about it.”

This quote reflects a finding from our content analysis: that forms included a number of tick-boxes that patients had to initial. Participants thought that these tick-boxes prompted discussion of, and reflection on, the featured issues, and by virtue of this, they might have increased the likelihood that patients were making choices that were autonomous.

Since forms were a consistent way for HCPs within each service to record information, they made it clearer to different HCPs within that service what agreements the patient had made (FG1P4). In this way, forms could be helpful for testing family members seen in the same service later on (as long as the patient had ticked ‘agree’ or initialled the statement about using test results to benefit relatives). HCPs who had decided to help relatives thought that forms would protect them against litigation;

FG16P3 “[We’re not] breaching confidentiality then, because [we’]ve got permission.”

Nonetheless, participants also acknowledged that the protective value of forms might be an illusion;

FG10P1 “It’s *like* a legal document but it’s not legally verified: it’s just for our peace of mind.”

P31 “You may think the [NHS] organisation would require it to protect them, but I’m not sure written consent would achieve that.”

In sum, participants thought that their forms were sometimes useful for helping patients to think carefully about tests and for facilitating the use of information to benefit family members. Nevertheless, they also thought that forms constrained good practice in several ways, and that they often did nothing to improve it. We summarise these issues in Fig. 6 and discuss them in the next section.

##### HCPs views on how forms constrained, or had limited use in, facilitating, good practice

In this section, we focus more specifically on the overlapping ways that forms were perceived to have restricted value or to be problematic: their limited use for benefiting relatives and for facilitating consent for IFs/VOUS; the burden of tick-boxes; and their potential to constrain the scope of practice by asking for overly-specific details.

While forms could help to make clear whether HCPs could use a test result from one patient to benefit family members later seen in their service, the forms were less helpful when the family member was seen elsewhere. The reason for this was that different services had different expectations and standards about the specific wording against which the patient should have signed, and thus whether they considered a completed form valid. HCPs explained they had to chase patients to fill out extra forms, which was distressing for those who wanted the clinical encounter to be finished [FG8]. This finding is particularly notable because consent forms should document a process whose whole purpose is to protect patients from harm. Participants also explained that the forms themselves, especially ones made long and complex by the number of items to read and tick, frustrated patients and were unacceptably burdensome. This weighed against the perceived benefit that forms might provide patients with a sense of control.

Interestingly, participants thought the way forms addressed the issue of using information to benefit relatives was particularly unclear on their forms and caused patients to worry;

FG8P2 “Our [form] is very much longer and more complicated than anyone else’s. I’m sure patients think, ‘oh my god’ when they see it, the amount of words. People often question the wording: are we going to write to their doctors first? Are we going to be writing to [or] contacting their family members? There’s a bit of anxiety.”

FG8P4 “It’s only because we’re asking them to tick something that it becomes a major issue. They think there’s a hidden agenda…that we’re going to find all their family members.”

Regarding IFs and VOUS, participants wondered whether and how forms should integrate discussion, and decisions made, about these findings, including whether they should incorporate categories of IFs they wanted fed back. One participant, who was resistant to forms getting longer (i.e. incorporating more tick boxes), wondered whether in future forms would *have to* mention these findings, because other areas of medicine had set a precedent for documenting consent on lengthy forms when the intervention could lead to several outcomes;

P31 “We [seek written consent] for taking photos of a child because we want to define for the family what we might use those photos for. There is a parallel there with what the blood might be tested for.”

However, generally, participants thought forms that attempted to integrate IFs/VOUS would become too long and too “complicated, with different scenarios” [P23]. They would be “overwhelming” for patients [P24] and make signing the form a disjointed part of the overall consultation—a bureaucratic exercise in an otherwise dialogical encounter. Some participants thought the number of tick boxes meant forms were *already* too long, hindering the flow of the consent process [P31], and making discussions with patients less individualised;

FG8P2 “[Forms are already] unnecessarily long, laborious, wordy, in the smallest print, complicated, [with] umpteen questions.”

Participants worried that longer, more complicated, forms would “eat into” (P1) the more important and tailored consent discussion, thereby constraining ethical practice;

FG6P3 “Most patients don’t know they’ve had a genetic test, even though they’ve signed a form. It’s sometimes not worth the paper it’s written on. It’s more important that that patient actually understands, that you’ve communicated that to them.”

Participants thought that this consent discussion, and not a signature on a form, determined the validity of consent.

Another related question about the consent process was how specific to be on forms, for example regarding what genes to test. Participants explained that being too broad caused patients to worry that HCPs might “go checking something else” [FG10P5]. However, writing something specific made HCPs question whether they had to seek fresh consent, for example, to ethically and legally test a different but potentially relevant gene;

FG8P4 “You don’t want such tight consent that you can’t use it”

Participants furthermore worried that patients might mistakenly perceive a named gene as a diagnosis;

FG7P3 “[Testing] can be ongoing: the reason to not discuss that [possible] diagnosis first is because [parents]’ll go straight on the internet, look it up, join a support group and then it’s not [the right diagnosis.]”

Dilemmas about whether to be specific or broad also related to HCPs’ perceptions about which findings they could feed back to patients;

P27 “We specify the condition we’re testing for, or the genes relating to [it]. You’d expect therefore only to feed back results relating to that condition. To give them the IF is outside of that written consent.”

In sum, while consent forms were useful to some extent for structuring discussion, the process of completing the form could distress and frustrate patients and crucially, there were concerns that the content of the forms could shape the scope of practice. For these reasons, participants felt that consent processes led HCPs to “tie [them]selves up in knots” [FG8P4].

## Discussion

### Summary of findings

This study is the first to investigate the way UK genetic services seek consent for genetic testing. We comprehensively and systematically analysed all clinical consent forms and explored HCPs’ views about their use. Our mixed-methods analysis has identified that consent forms might help patients reflect on decisions in the consent process and make these decisions clear to other HCPs seeing the same family. However, it has also identified several problems: consent forms imply that HCPs take an individual approach to confidentiality despite national guidelines recommending otherwise; HCPs perceive forms to have limited use for addressing IFs/VOUS; and forms offer multiple tick-boxes that may do little to enhance autonomy and may constrain decision making, for example, around what findings to feed back. We discuss these issues in more detail now.

### The individual approach to confidentiality on forms

Through our content analysis, we found that many genetic services presented patients with a choice over whether test results could be used to benefit relatives. The forms’ authors may have decided that offering a choice is ethically preferable on the grounds that patients should have control over how genetic information ‘generated’ by their test is used and that *all* information should be kept confidential to individuals. Furthermore, they might consider the JCGM’s framing of the issue as a *statement* rather than a choice to be a type of nudging, whereby a default rule is set that automatically opts people into a particular option (one that prioritises their welfare over their liberty, on the assumption that people tend not to opt out of default settings) [19]. This is particularly relevant to consider as Wouters et al. have recently argued that HCPs should use a ‘moral accountability nudge’ to direct patients to tell their relatives about a genetic risk [20]. Nudging is controversial and has been criticised for “surrender[ing] too much libertarian ground to paternalism” [21]. We would contend that the use of a statement to encourage the use of information to benefit relatives is not a type of nudging—not least because it does not prioritise an individuals’ welfare over their liberty. Moreover, it does not attempt to influence an individual’s behaviour. Rather, it considers it inappropriate to give an individual person the right of veto over the disclosure of information—which, although discovered in them, might be familial in nature—with those who could benefit from knowing.

We did not present an analysis of the specific phrasing of the familial issue on individual consent forms, but we notably found that participants thought this aspect of the form worried some patients and made them wonder whether HCPs would share their personal clinical information (e.g., breast cancer diagnosis) with others. This worry might suggest that the difference between this information and familial genetic information (e.g., a BRCA1 mutation) is not always clear to patients—and perhaps not to HCPs. Research does however show that patients support HCPs adopting familial confidentiality, as long as HCPs make clear they are doing so at the time of testing [22, 23].^2^ Patients generally want family members to know their risks and to have the option of testing, but do not always tell them about the risk themselves for various reasons, for example because they do not know who to tell and what to tell them [22, 23]. Nevertheless, the upcoming revised JCGM guidelines should address the issue that the mention of sharing information might make some patients anxious. Specifically, the guidelines could make the differences between personal and familial genetic information clearer on consent forms.

### Feeding back IFs and the over-use of tick boxes

All services used forms that were more complicated than the JCGM form: although none offered categories of IFs as suggested by Ayuso et al. [3] and others, they did offer choices/tick-boxes about other things and included (sometimes several) additional statements. This finding makes it more significant that HCPs worried about whether they had to adhere to the specifics on the form for practice to be ethical and legal: the greater number of specific choices that are included on forms, the greater number of things there are to constrain the scope of their practice. Regarding the feedback of IFs, Pereira et al. [24] have recently cautioned against using consent forms to shape practice, but point out that forms are already taking on this role. For example, research ethics committees often advise that results from large-scale genomic studies be given to participants only if the original study’s consent form mentioned such feedback. The problem here is that consent documents do not always anticipate future possibilities, such as the prospect of identifying IFs and providing these results. These authors argue that it would be more ethical to make feedback decisions on a case-by-case basis, which might include considerations about the potential clinical significance of the finding and what the patient might want to know. The revised JCGM guidelines should consider whether decision-making on a case-by-case basis is more appropriate than offering categories of IFs on clinical consent forms.

This consideration is especially important, because not only can forms with several tick-boxes and choices constrain practice, they might also frustrate and distress patients, as our participants explained. Evidence from wider genetics research and biobanking shows that long forms, especially if people have to complete them quickly, can be intimidating and difficult to read and understand [25–27]. Indeed, we found aspects of the forms difficult to interpret ourselves. Long forms can lead to acquiescence bias, i.e. taking mental shortcuts and agreeing to all options [28]. Offering several complex choices on forms moreover assumes that all patients are equipped to make these choices, which infringes upon justice and fairness for patients who want more directive advice [11]. Overall, trying to include all issues that might arise from genetic testing could weaken the process of consent [29].

### Why genetic services use these forms

Several overlapping factors could explain why forms have taken on this character, and why services adopt an individual, rather than a familial, approach to confidentiality. One reason is that in genetic medicine, clinical practice and research have always had a close relationship [30], which has raised questions about what exactly consent forms ought to mention. Those who decide might have tended towards the more ‘cautious’ end of the spectrum, designing clinical forms to be more like the comparably formalised research forms. Such decisions might be influenced by the ethos in Western health services, which is characterised, according to O’Neill [31], by a fear of liability and a prevailing ‘audit culture’—a perception that everything must be documented to show that regulations are being met. Running through all of this is the importance placed on individual autonomy in Western medicine and the unevidenced perception that clinical consent requirements, and the provision of increasing choice, can enhance it. O’Neill argues on the contrary: offering increasing choice and emphasising documentation is not a route to enhancing desirable values,  including transparency and trust. Instead, it “subjects patients to the standards of accountability of [an] audit agenda. [And this] is ethically questionable” (p156).

### Limitations

It might be that in other healthcare services, particularly in non-Western countries, the consent process is different. Indeed, our study was limited to the UK. This was for specific reasons—the country has a national health service and national guidelines, thus some consistency might have been found. We nevertheless call for research that can make judgements about practices elsewhere. Another limitation of our study is that, despite targeting our recruitment, our qualitative arm did not include participants from all the services in the UK that offer genetic tests. Nonetheless, we do not claim our findings to be generalisable. Our findings are, however, ‘transferable’: we have made our research context explicit so that other services, within the UK and elsewhere, can determine the extent to which the findings apply to their settings [18].

### Implications for practice

At a time when healthcare services worldwide are integrating next-generation tests, and where there is increasing integration of research and clinical practice, we must consider how consent can serve patients’—and relatives’— interests best. In addition, since consent forms can influence, and be influenced by, approaches to consent, it is important to invest careful thought into the way they are designed and used. We recommend that

1. Patients not be given an absolute right of veto over familial genetic information that could benefit family members, and that consent forms should make this familial approach clearer.
2. Consent forms be streamlined and simplified and do not attempt to incorporate complex categories of IFs.
3. Professionals do not use the specific details on consent forms in place of their professional judgement when making decisions about benefitting relatives and feeding back IFs.
4. Consent forms, at least within each country, be standardised, to facilitate the sharing of information between services, which will in turn increase patient equality.

## Conclusion

We have investigated the approach to and documentation of consent for genetic tests in current UK practice, paying attention to whether and how familial confidentiality and IFs/VOUS are addressed, and whether forms would suggest that the consent process is becoming, as Dunn worries, “a tick-box exercise...align[ing practice] with prevailing professional requirements” [14]. Insofar as its tick-box aspect, Dunn’s concern bore out. In fact, our study showed that consent processes did not align with guidelines from the relevant UK professional society—the JCGM. Instead, they aligned with a *perceived* professional requirement to regard familial genetic information as confidential to individuals and to offer patients multiple choices on the flawed assumption that this enhances autonomy. Consent forms, according to HCPs, facilitated good practice to some extent, but they could also constrain good practice and be used in place of professional judgement. We make several recommendations about the way consent should proceed, including that forms should not provide multiple tick boxes, for example to provide choices about IFs. We moreover encourage further debate to ascertain good practice. Without this, we run the risk of replacing valid consent processes with ever-longer forms that do nothing to improve consent and to benefit patients and their relatives.

## Footnotes

1 This was not to castigate services not using the JCGM form, but because the guidance was published in 2011 and was compiled by UK royal colleges and societies, raising a question about whether the guidelines had changed practice.

2 There might still be good reasons for HCPs not to use genetic information in this way—for example, if the risk of a relative inferring the patient’s identity is high and, because of some characteristic of the patient and relative’s relationship, inference could lead to patient harm. It is important for HCPs to make such considerations when deciding whether to disclose information and we lay out this argument, in detail, elsewhere [19].
